# Insulin Resistance in Long COVID-19 Syndrome

**DOI:** 10.3390/jpm14090911

**Published:** 2024-08-28

**Authors:** Dana Emilia Man, Minodora Andor, Valentina Buda, Nilima Rajpal Kundnani, Daniel Marius Duda-Seiman, Laura Maria Craciun, Marioara Nicula Neagu, Iulia-Stefania Carlogea, Simona-Ruxanda Dragan

**Affiliations:** 1 Department VI—Cardiology, University Clinic of Internal Medicine and Ambulatory Care, Prevention and Cardiovascular Recovery, “Victor Babes” University of Medicine and Pharmacy, 3000041 Timisoara, Romaniaknilima@umft.ro (N.R.K.); maria-laura.craciun@umft.ro (L.M.C.);; 2 Research Centre of Timisoara Institute of Cardiovascular Diseases, “Victor Babes” University of Medicine and Pharmacy, 3000041 Timisoara, Romania; 3 Discipline of Medical Semiotics II, Department V—Internal Medicine—1, “Victor Babes” University of Medicine and Pharmacy, 3000041 Timisoara, Romania; 4 Department I, Faculty of Pharmacy, University Clinic of Clinical Pharmacy, Communication in Pharmacy, Pharmaceutical Care, “Victor Babeş” University of Medicine and Pharmacy, 2 Eftimie Murgu Square, 300041 Timisoara, Romania; 5 Faculty of Bioengineering of Animal Resources, Discipline of Physiology University of Life Sciences “King Mihai I” from Timișoara, University of Life Sciences “King Mihai I”, 300645 Timișoara, Romania; 6 Faculty Medicine, “Victor Babeş” University of Medicine and Pharmacy, 2 Eftimie Murgu Square, 300041 Timisoara, Romania

**Keywords:** SARS-CoV-2, COVID-19 syndrome, diabetes mellitus type 2, glucose impairment, inflammation, biomarkers

## Abstract

**Background:** The COVID-19 pandemic has caused severe health issues worldwide and contributed to huge financial losses. Key comorbidities linked to an increased risk of severe COVID-19 and higher mortality rates include cardio-metabolic disorders such as type 1 and type 2 diabetes mellitus (T1DM and T2DM), atherosclerotic cardiovascular disease, chronic kidney disease, hypertension, heart failure, and obesity. The persistence of symptoms even after the acute phase is over is termed long COVID-19 syndrome. This study aimed to evaluate the relationship between long COVID-19 syndrome and the development of insulin resistance in previously non-diabetic patients. **Methods:** A prospective observational study was performed on 143 non-diabetic patients who had tested positive for SARS-CoV-2 infection by a PCR test and were hospitalized in our hospital between January 2020 and December 2022. The clinical and para-clinical data at 0, 4, and 12 months of hospital admission for post-COVID-19 infection follow-up was collected and labeled as t0, t4, and t12. Blood glucose, insulin, and C-peptide levels were measured at the beginning and further at 2, 5, 10, and 30 min after the intravenous arginine stimulation test. Similarly, BMI was calculated, and hs-CRP and ESR levels were noted. The results obtained were statistically analyzed. **Results:** More than one-third (30.7%) of the included patients developed long COVID-19 syndrome. It was found that 75% of patients with long COVID-19 hospitalized in our clinic developed diabetes within a year of acute infection with COVID-19; therefore, it can be said that the presence of long COVID-19 is a major risk for an altered metabolic status, which can cause diabetes. When comparing the glycemia levels (106 mg/dL) with the BMI at t0, t4, and t12 time intervals, the p-values were found to be 0.214, 0.042, and 0.058, respectively. Almost 62% of the patients having BMI > 30 kg/m^2^ were found to have an increase in blood glucose levels at 1 year. Similarly, insulin resistance was noted during this interval. A negative correlation of 0.40 for hsCRP and 0.38 for ESR was noted when compared with acute infection with COVID-19. **Conclusions:** The association between long COVID-19 and insulin resistance highlights the varied and widespread impacts of SARS-CoV-2 infection. Addressing the complexities of long COVID-19 requires a holistic strategy that encompasses both respiratory and metabolic considerations, which is crucial for enhancing the well-being of those enduring this persistent condition.

## 1. Introduction

The Severe Acute Respiratory Syndrome Coronavirus 2 (SARS-CoV-2), the cause of the COVID-19 pandemic, has had a profound impact on global health and the economy. As of April 2021, the virus infected over 120 million people worldwide and resulted in more than 2.8 million deaths [1]. The pandemic has disproportionately affected the elderly, individuals from lower socioeconomic backgrounds, ethnic minorities, and those with certain chronic health conditions [2]. Common comorbidities that have been consistently linked with an increased risk of severe COVID-19 and higher mortality rates include cardio-metabolic risk factors such as type 1 diabetes mellitus (T1DM), type 2 diabetes mellitus (T2DM), rhythm disturbances, atherosclerotic cardiovascular disease, chronic kidney disease, hypertension, heart failure, and obesity [3,4,5].

As the world continues to grapple with the effects of the COVID-19 pandemic, a growing body of evidence suggests that some individuals experience lingering symptoms long after recovering from the acute phase of the illness. This condition, commonly known as “Long-COVID-19” or “post-acute sequelae of SARS-CoV-2 infection” (PASC), has been associated with a variety of persistent symptoms, including fatigue, dyspnea, cognitive impairment often referred to as “brain fog”, joint pain, and respiratory issues [6]. More precisely, if these symptoms persist post-infection for more than 30 days, it can be labeled as long COVID-19 [6]. These symptoms can affect multiple organ systems and can exhibit serious complications.

Chronic inflammation is a hallmark of long COVID-19 and is thought to contribute to many of these persistent symptoms. The exact mechanisms driving long-term inflammation after COVID-19 are still under investigation, but several factors are thought to play a role [7,8,9,10]:

- Immune System Dysregulation: COVID-19 can cause prolonged activation of the immune system, leading to a state of chronic inflammation.
- Viral Persistence: Some researchers suggest that viral fragments remaining in the body may continuously trigger an immune response.
- Autoimmunity: COVID-19 might trigger autoimmune responses, leading to ongoing inflammation.

### Impact of COVID-19 on Metabolic Health 

Following the acute phase of SARS-CoV-2, many individuals suffer from long COVID-19, which includes a range of symptoms [11]. Hyperglycemia often occurs in patients with SARS-CoV-2, regardless of their diabetes status before admission [12]. Diabetes is a complex chronic metabolic disorder, and in the case of type 2 diabetes mellitus, it involves beta-cell dysfunction amidst insulin resistance. SARS-CoV-2 can trigger new-onset hyperglycemia, exacerbate pre-existing diabetes, or even cause hyperglycemic emergencies. Studies have linked the degree of hyperglycemia to the severity of SARS-CoV-2 infection, as high glucose levels promote viral replication, leading to cytokine production and T-cell dysfunction [13].

The pathogenesis of hyperglycemia in SARS-CoV-2 infection is multifaceted. The angiotensin-converting enzyme 2 (ACE 2), which is the primary receptor for SARS-CoV-2, is present in various human cells, including pancreatic islet cells. SARS-CoV-2 attaches to the ACE 2 receptors on pancreatic islet cells, causing transient damage and insulin deficiency. Infection with SARS-CoV-2 leads to an increase in inflammatory markers, particularly acute-phase reactants. These reactants might directly contribute to the destruction of pancreatic fat cells and induce insulin resistance through mechanisms like ketogenesis and adipocyte dysfunction [14]. This insulin resistance heightens lipolysis and elevates circulating free fatty acids, exacerbating hyperglycemia, especially during the cytokine storm phase [15].

The link between obesity and the occurrence of diabetes, particularly after COVID-19, has become a significant area of research and concern. The COVID-19 pandemic has highlighted and exacerbated several health issues, including the relationship between obesity, diabetes, and the virus itself. The virus can infect pancreatic beta cells, which are responsible for insulin production, potentially leading to acute hyperglycemia (high blood sugar levels) and contributing to the development of diabetes. The stress response induced by severe illness, including COVID-19, can lead to insulin resistance and hyperglycemia. Cytokine release during SARS-CoV-2 infection is thought to potentially trigger metabolic changes by disrupting glucose homeostasis. This could occur through several mechanisms: revealing pre-existing conditions, stress responses, hypoxic lung injury, direct damage to pancreatic cells, and steroid-induced hyperglycemia. Patients with COVID-19 often exhibit decreased lymphocyte counts and elevated neutrophil levels. Additionally, pro-inflammatory markers like high C-reactive protein (CRP), procalcitonin, and ferritin are commonly elevated in individuals with both COVID-19 and diabetes. These inflammatory responses are indicative of the body’s heightened immune activity against the virus and are also associated with metabolic disturbances observed in diabetes [16]. Research suggests that the immune dysregulation in COVID-19, characterized by lymphopenia and neutrophilia, contributes to the severity of the disease. Elevated CRP, a marker of inflammation, is frequently observed in severe COVID-19 cases and is linked to poor outcomes. Similarly, procalcitonin, which is typically elevated in bacterial infections, is also found to be raised in COVID-19, indicating a severe systemic inflammatory response. Ferritin, an acute-phase reactant, is another marker that is significantly elevated in severe COVID-19 cases and is associated with cytokine storm syndrome, reflecting the hyper inflammatory state [17]. In diabetes, chronic inflammation is a common underlying feature. The elevated levels of CRP, procalcitonin, and ferritin in diabetic patients suggest that pre-existing inflammatory states may exacerbate the inflammatory response to COVID-19, leading to worse outcomes. This interplay between diabetes and COVID-19 highlights the importance of monitoring and managing inflammatory markers to mitigate complications in affected patients [18]. Hence, this can precipitate the onset of diabetes in individuals with pre-existing risk factors, such as obesity [19].

One emerging aspect of long COVID-19 has garnered attention due to its potential connection to insulin resistance. Patients previously having insulin resistance can suffer severe complications of the COVID-19 infection, while on the other hand, insulin resistance can develop in a previously healthy individual post-long COVID-19 syndrome [20]. Insulin resistance is a metabolic condition in which the body’s cells become less responsive to the effects of insulin, a hormone crucial for regulating blood sugar levels. When cells resist insulin’s signals, glucose is not efficiently absorbed by the cells, leading to elevated blood sugar levels. As mentioned earlier, this condition is often associated with type 2 diabetes, obesity, and metabolic syndrome, which further gets complicated when there is an acute or chronic inflammatory state [21].

Worldwide studies are being conducted for better insight into the connection between long COVID-19 and insulin resistance. A notable study published in ‘Diabetes, Obesity and Metabolism’ [22] revealed that people who had recovered from COVID-19 showed indications of insulin resistance, irrespective of their diabetic status before infection. This finding implies that the virus could directly affect insulin sensitivity, causing metabolic issues even in those without diabetes. Additionally, the immune response to the initial COVID-19 infection may contribute to the development of insulin resistance. Long COVID-19 often involves chronic inflammation, which is known to be a factor in the emergence of insulin resistance. The sustained activation of the immune system might interfere with insulin’s normal functions and its pathways, resulting in metabolic imbalances [23,24].

The aim of this study was to see if non-diabetic patients suffering from long COVID-19 syndrome developed insulin resistance over time or not.

## 2. Materials and Methods

A prospective observational study was performed between January 2020 and December 2022 at the Cardiovascular Rehabilitation Center of the Institute of Cardiovascular Diseases, Timisoara, Romania. Out of the 1980 total patients admitted during this time interval, 143 cases were selected for the study. Patients who tested positive for SARS-CoV-2 infection on PCR tests were included in the study. Patients who were previously diagnosed with diabetes mellitus type 1 or type 2, insulin resistance, other metabolic disorders, chronic respiratory insufficiency, asthma, pathologies resulting in chronic inflammatory states, liver or pancreatic diseases, or patients who had undergone recent surgeries were excluded. Long COVID, also known as post-acute sequelae of SARS-CoV-2 infection (PASC), refers to a range of symptoms that can persist for weeks or months after the acute phase of a COVID-19 infection has resolved. Common symptoms include fatigue, shortness of breath, cognitive dysfunction (often referred to as “brain fog”), muscle pain, joint pain, chest pain, cough, difficulty sleeping, headache, heart palpitations, loss of taste or smell, depression or anxiety, fever, and symptoms that worsen after physical or mental activity.

### 2.1. Clinical Variables and Laboratory Parameters

Epidemiological factors such as gender, age, body mass index, smoking status, and hypertension, along with clinical and laboratory data, were analyzed. This included an evaluation of cardiovascular risk factors, notably the SCORE risk, as well as a review of personal pathological history and comorbidities. Renal and liver function tests; lipid profiles; and glycemic profiles, including insulin levels, Homeostasis Model Assessment of Insulin Resistance (HOMA-IR), and triglyceride/glucose (TyG) index, were assessed, along with other inflammatory markers. Biochemical analyses were conducted using fasting serum samples from patients, collected at specific time points, and stored at −80 °C. Serum proinsulin and HbA1c levels at baseline were measured using ELISA kits. Basal blood glucose levels were determined using a colorimetric assay on serum samples collected in potassium oxalate/sodium fluoride tubes. Serum samples obtained from participants during the arginine stimulation test were collected at each designated time point (t0-t4-t12). These time points refer to their first admission to our hospital when they tested positive for the first time (t0), while t4 and t12 refer to their follow-up admissions to our hospital at 4 and 12 months after the first admission due to COVID-19 infection. Further, these were analyzed for insulin, C-peptide, and glucagon concentrations. All the tests were performed while respecting the manufacturer’s guidelines. 

### 2.2. Hormone Level Assessment

Insulin and C-peptide production was measured using an intravenous arginine stimulation test. A catheter was placed into the patient’s arm at the antecubital vein. To maintain the catheter’s patency when not in use, a slow infusion of 0.9% saline was administered. Baseline samples were collected at the 0 min mark. A maximum dose of arginine hydrochloride (5 g) was administered intravenously over 45 s. Samples were collected at 2, 5, 10, and 30 min after injection. For fasting glucose, the Acute Insulin Response to Glucose (AIRglucose) was calculated as the average of the three highest insulin readings at 2, 5, and 10 min, minus the baseline insulin value. The ratio of fasting insulin to proinsulin was used to assess the beta cell function. Insulin resistance was determined using the HOMA-IR equation: fasting insulin (mIU/mL) × fasting glucose (mmol/L)/22.5. 

### 2.3. Statistical Analysis

The characteristics of patients included in the study were presented as actual numbers with percentages (%) for categorical variables and mean with standard deviation for continuous variables. We used independent *t*-tests and chi-square analyses to compare the clinical characteristics of the two groups. A two-sided *p* < 0.05 was considered statistically significant. Age and sex were included in the multivariable logistic regression analysis performed for each clinical outcome.

### 2.4. Ethics Approval and Patient Informed Consent

Before commencing the study, ethics approval was obtained from the Ethics Committee for Scientific Research of the Cardiovascular Institute of Timisoara (ref. nr. 2193/25 March 2024), University Emergency Hospital. The study was carried out in accordance with the Declaration of Helsinki. Written informed consent was obtained from all patients as a part of routine admission to our tertiary University hospital for future research and study purposes.

## 3. Results

We calculated, in the studied group of 143 hospitalized patients with the moderate/severe form of COVID-19, the state of insulin resistance at the time of inclusion, at the 4-month follow-up, and at the 12-month follow-up after the hospitalization with a post-acute episode of COVID-19. The general characteristics were noted (Table 1). We further compared the results based on BMI if the said patients were obese or not. The results are presented in Table 2. A statistical test to compare the proportions of the patients developing diabetes (glycemia > 106 mg/dL) was applied using a unilateral zero hypothesis of “glycemia in obese patients is higher than in non-obese”, with a confidence level of 95%. We can observe a much higher incidence rate of diabetes in obese patients than in those without obesity, the differences between the groups being significant at discharge at 4 months after the acute episode of COVID-19 and close to significant at 12 months. 

It can also be observed that the number of non-obese patients in our group was lower compared to those suffering from obesity. This illustrates the fact that the patients who required hospitalization for moderate or severe forms of COVID-19 were predominantly obese.

**Figure 1:**
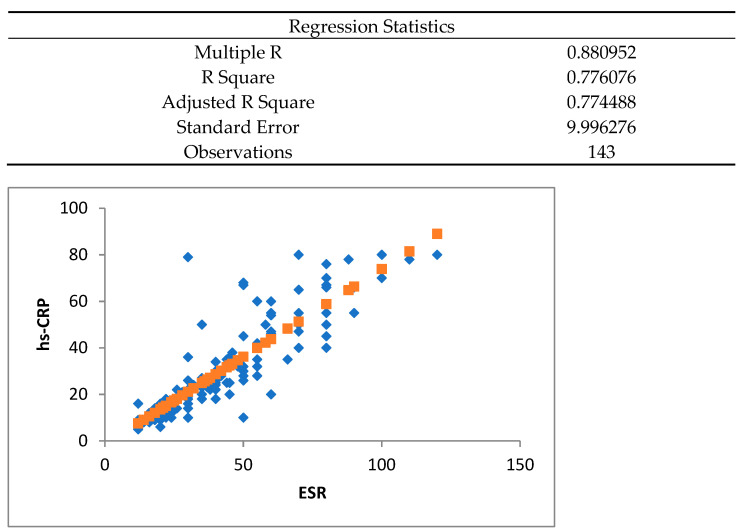
Correlation between ESR and hsCRP (ESR: erythrocyte sedimentation rate; hsCRP: high-sensitivity C-reactive protein).

In the group studied by us, in the 143 patients discharged after the acute episode of COVID-19, persistent inflammatory factors were determined at discharge at 4 months and 12 months. The sedimentation rate of erythrocytes (ESR) and highly sensitive C reactive protein (hsCRP) were determined. The values for these were considered elevated if they exceeded the values of 12 mm/h for ESR and 3 mg/L for hsCRP. Both markers used for inflammation were well correlated with each other (Figure 1), so we used—for the diagnosis of long COVID-19—patients who showed increased ESR or hsCRP values at the 4-month visit and the 12-month visit, respectively, along with symptoms like fatigue, shortness of breath, cognitive dysfunction (often referred to as “brain fog”), muscle pain, joint pain, chest pain, cough, difficulty sleeping, headache, heart palpitations, loss of taste or smell, depression or anxiety, and fever.

Following this classification, we divided our group into patients with long COVID (48 pts) and patients without long COVID (95 pts). The incidence rate of diabetes was examined in these patients (Table 3).

The χ^2^ test was performed to compare the results. It was found that 75% of patients with long COVID-19 hospitalized in our clinic developed diabetes within a year of acute infection with COVID-19, compared to 55.8% of those who did not develop long COVID-19. The difference between the groups is statistically significant; therefore, it can be said that the presence of long COVID-19 is a major risk for an altered metabolic status, which can cause diabetes.

**Figure 2:**
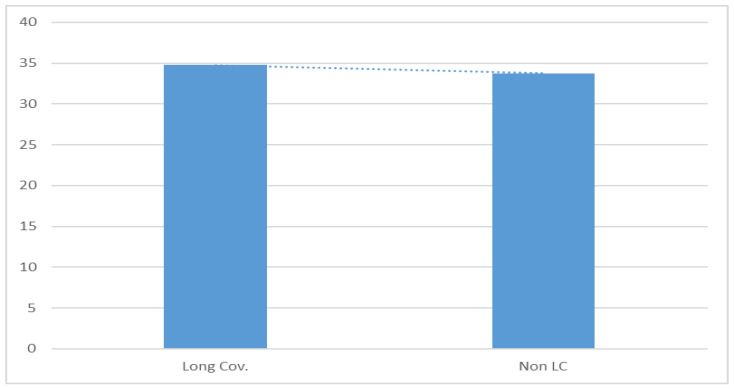
Correlation between the inflammatory status and metabolic status after COVID-19 acute infection. Long Cov: long COVID-19; Non LC = non-long COVID-19.

There was a correlation between obesity (BMI > 30 kg/m^2^) and the occurrence of long COVID-19 in the patients of our group. To our surprise, obesity was not more common in patients with long COVID-19 compared to those without long COVID, with *p*-value = 0.277. Thus, there is no significant difference between the groups (Table 4 and Figure 2).

The correlation between the degree of COVID-19 damage, measured by increased values of inflammatory factors at discharge, when the patient was considered cured of the acute phase of COVID-19, and the metabolic status of the patients, measured by the fasting blood glucose, the triglyceride–glucose index (TyG), the Homeostatic Model Assessment for Insulin Resistance (HOMA-IR), and insulinemia, is shown in Table 5.

The presented results include the Pearson linear correlation between two pairs of variables after eliminating four cases of outliers that exceeded 5xIQR for HOMA or TyG.

In the patients from our group, there is a negative correlation of 0.40 for hsCRP and 0.38 for ESR, which does not show a very close correlation. 

## 4. Discussion

Recent research findings indicate that individuals with SARS-CoV-2 infections experienced a decline in their glucose and lipid metabolism, even in the absence of any pre-existing metabolic conditions [25]. Montefusco et al. [17] noted elevated levels of insulin in patients with SARS-CoV-2 infections and observed an increased concentration of C-peptide in those who had acute respiratory distress syndrome (ARDS). Significantly, the insulin resistance observed could persist even after the virus is eliminated, suggesting a possible long-lasting health issue for SARS-CoV-2 patients. These results highlight the need for increased monitoring and potential interventions during the follow-up care of individuals who experience metabolic disruptions due to SARS-CoV-2 infection. In addition to pre-existing cardio-metabolic conditions that elevate the risk of severe COVID-19 and mortality, patients hospitalized with COVID-19 face a heightened likelihood of experiencing acute cardiorenal complications. A meta-analysis encompassing 44 studies involving 14,866 COVID-19 cases, conducted in 2020 [26], revealed that acute cardiac injury affected 15% of patients (with a 95% confidence interval ranging from 5% to 38%). Venous thromboembolism occurred in 15% of patients (with a 95% confidence interval ranging from 0% to 100%), and acute kidney injury manifested in 6% of patients (with a 95% confidence interval ranging from 1% to 41%). Many of these acute complications can persist as part of the long-term effects of COVID-19, commonly known as long COVID-19.

Furthermore, a study conducted in the UK, involving 201 individuals with an average age of 44 years, which incorporated detailed MRI assessments, found that at a median follow-up of 140 days after COVID-19 infection, 98% of participants reported fatigue, 88% experienced muscle aches, and 87% had breathlessness. Alarmingly, mild organ impairments were evident in the heart (32%), lungs (33%), kidneys (12%), liver (10%), and pancreas (17%), and 25% of individuals exhibited multi-organ impairments [27]. Consequently, in populations of young individuals with low risk, approximately two-thirds still experience ongoing harm to at least one organ four months following the onset of SARS-CoV-2 symptoms, suggesting significant long-term health consequences for these patients. 

Furthermore, many symptoms overlap or mimic other diseases. A careful evaluation is required to label the patients as suffering from long COVID. To rule out our disparities in diagnosis and to obtain a clear understanding of this newly diagnosed condition, the term “Long COVID” was proposed to be included in the ICD-10 nomenclature coding [7]. 

Studies have reported an increased incidence of new-onset diabetes among individuals recovering from COVID-19. This is thought to be partly due to the inflammatory effects of the virus on the pancreas and other metabolic organs. Chronic inflammation can damage pancreatic beta cells, impairing insulin production and contributing to the development of diabetes.

### 4.1. Relationship between Obesity and Long-COVID

Obesity is a significant risk factor for severe COVID-19 outcomes, including hospitalization, ICU admission, and mortality. This is due to factors such as chronic inflammation, impaired immune response, and metabolic complications associated with obesity.

An explanation for these results is that obesity is highly prevalent in the general population, particularly in regions with high COVID-19 infection rates. This means that both long COVID-19 and non-long COVID-19 patients are likely to include a significant number of obese individuals. Many studies on long COVID focus on patients who were hospitalized with severe COVID-19. Since obesity is a risk factor for severe disease, a high percentage of these patients are likely to be obese, whether or not they develop long COVID-19.

The precise causes linking cardio-metabolic disorders to increased mortality from severe COVID-19 remain unclear. It has been observed that acute respiratory viral infections, including COVID-19, can cause temporary insulin resistance in those with type 1 and type 2 diabetes mellitus. Additionally, high blood sugar levels are independently associated with a heightened risk of severe COVID-19 and increased mortality in individuals with type 2 diabetes [28]. A widely accepted hypothesis suggests that patients with chronic metabolic inflammation are more susceptible to an overproduction of cytokines, often referred to as a cytokine storm. This excessive release of inflammatory cytokines could potentially lead to multi-organ failure [28]. The primary receptor for SARS-CoV-2 entry is the angiotensin-converting enzyme 2 (ACE2). When SARS-CoV-2 attaches to ACE2 receptors in the pancreas, it can harm the islet cells and diminish the pancreas’s ability to secrete insulin, thereby responding inadequately to the resulting high blood sugar levels [28].

There is a good correlation between hsCRP and HOMA-IR and insulin levels. The correlation between hsCRP and HOMA-IR, as well as insulin levels in the blood, is well-documented and underscores the role of inflammation in the pathophysiology of insulin resistance. Elevated hsCRP levels reflect chronic low-grade inflammation, which interferes with insulin signaling and contributes to higher fasting insulin and glucose levels, leading to increased HOMA-IR scores. This relationship highlights the importance of addressing inflammation to manage and prevent insulin resistance and its associated metabolic disorders.

However, there was not as good of a correlation with TyG, which is considered in the literature to be a very accurate marker of insulin resistance. Patients, after contracting SARS-CoV-2 infection, even if asymptomatic or mild, have an increased risk of presenting T2DM, as SARS-CoV-2 infection is associated with impaired glucose metabolism.

Patients, after contracting the SARS-CoV-2 infection, even if asymptomatic or mild, have an increased risk of presenting T2DM, as SARS-CoV-2 infection is associated with impaired glucose metabolism.

Several other potential pathophysiological processes have been suggested, such as elevated levels of tissue-specific enzymes, changes in ACE2 receptor expression, immune system dysregulation, pulmonary and endothelial impairment, systemic inflammation, and hypercoagulability. Furthermore, heightened levels of anti-inflammatory biomarkers like C-reactive proteins, D-dimer, and IL-6 may play a role. In individuals with type 1 or type 2 diabetes mellitus, these pathophysiological disruptions could intensify the inflammatory cytokine storm response, potentially resulting in more severe COVID-19 manifestations [28]. A systematic analysis of eight retrospective cohort studies conducted in 2020 also revealed a correlation between excess body fat and an increased risk of severe illness and death in individuals infected with COVID-19 [29]. Most individuals with cardio-metabolic diseases also suffer from obesity and chronic low-level systemic inflammation. This could be a key factor connecting severe COVID-19 to insulin resistance, type 2 diabetes mellitus, hypertension, and cardiovascular diseases. Biomarkers important in establishing the prognosis of cardiovascular events witnessed in COVID-19 infections are considered to be miRNAs. A review conducted by Izzo C. et al. [30] demonstrated the pathophysiology behind the involvement of miRNA in the destruction process leading to CV events in COVID-19 patients. Future studies can pave the path to the development of new treatment regimens to minimize or exclude the cardiovascular damage caused by the coronavirus using miRNA-based strategies. 

Regarding the treatment of long COVID-19, it is crucial to manage risk factors like blood pressure, lipid levels, and obesity following a COVID-19 infection. Clinicians should also encourage enhancing physical function through lifestyle modifications and quitting smoking. There is substantial evidence that controlling risk factors such as blood pressure, dyslipidemia, and glucose levels can reduce both microvascular and macrovascular complications in the long-term care of patients with type 2 diabetes. Additionally, there is evidence supporting the long-term benefits of addressing multiple risk factors on kidney, cardiovascular, and mortality rates. We believe that these findings could similarly be relevant for individuals experiencing long COVID-19 [31].

The results from our study, when comparing inflammatory status and metabolic status after acute infection, can, however, be explained by the multifactorial nature of both inflammation and metabolic regulation, temporal differences in marker expression, individual variability, and the complexity of compensatory mechanisms. The impact of COVID-19 on metabolism and inflammation may differ in the acute phase versus the long-term recovery phase. TyG might be more reflective of long-term metabolic changes rather than acute inflammatory responses. Inflammatory markers and metabolic indices like TyG may peak at different times during and after the infection. Inflammation might resolve before significant changes in triglyceride and glucose levels become evident, or vice versa. 

The interplay between obesity and diabetes has been significantly impacted by the COVID-19 pandemic. Obesity increases the risk of severe COVID-19 outcomes, which can, in turn, exacerbate metabolic dysfunction and lead to the development of diabetes. The pandemic has also influenced lifestyle factors that contribute to obesity and diabetes risk, highlighting the importance of addressing these interconnected health issues [32]. Factors that contribute to poor outcomes in individuals with COVID-19 and long COVID-19 include obesity, high blood sugar, as well as cardiovascular and kidney diseases [33]. 

For the long-term management of individuals with long COVID-19, glucose-lowering medications that enhance metabolic function and offer additional benefits for the critical processes affected by COVID-19 are desirable. Regarding new treatment approaches, cardiovascular outcome trials in patients with type 2 diabetes have demonstrated the effectiveness of sodium-glucose cotransporter 2 (SGLT2) inhibitors and glucagon-like peptide 1 receptor agonists (GLP1RA). These drugs have shown benefits in weight management, blood sugar control, and cardiovascular events, including cardiovascular mortality, as well as in renal outcomes [34].

SGLT2 inhibitors not only reduce hospitalizations due to heart failure but may also lower the risk of death from non-cardiovascular causes. Most guidelines recommend SGLT2 inhibitors and GLP1RA as safe for clinical use during the pandemic. However, SGLT2 inhibitors should be discontinued in symptomatic SARS-CoV-2-infected individuals at risk of a disrupted fluid balance or (euglycemic) ketoacidosis. The DARE trial is currently assessing the effectiveness of dapagliflozin in hospitalized COVID-19 patients (NCT04350593). SGLT2 inhibitors and GLP1RA may potentially aid in the improvement of long COVID-19 through various mechanisms, including better control of high blood sugar, blood pressure, weight, oxidative stress, insulin resistance, and chronic low-level inflammation [31].

The advantages seen in COVID-19 patients treated with SGLT2 inhibitors extend beyond glucose regulation and encompass impacts on heart muscle metabolism, adipokine activity, and vascular functions [35]. Research has now also established that the cardiovascular and kidney advantages seen in individuals with type 2 diabetes mellitus (T2DM) who are treated with SGLT2 inhibitors are applicable to those without T2DM as well [35]. Considering the underlying mechanisms of COVID-19 and the documented advantages of SGLT2 inhibitors and GLP1RA, these treatments may offer unique benefits compared to other therapies for individuals with type 2 diabetes mellitus (T2DM) and long COVID-19 and potentially even for those without diabetes. Nevertheless, there is a critical need for randomized trials and practical studies to assess if the potential gains of strict multifactorial risk factor management and novel therapeutic approaches are effectively realized in everyday clinical settings for patients with long COVID-19.

Recognizing the link between long COVID-19 and insulin resistance is crucial for patient management. Healthcare providers should be cognizant of the potential metabolic impacts and consider screening for insulin resistance in those with persistent long COVID-19 symptoms. Early identification and treatment of insulin resistance can enhance long-term health outcomes and lower the risk of developing type 2 diabetes [36]. The results from our study clearly indicate that the chances of acquiring diabetes or carbohydrate metabolic disturbances increase; hence, it can be stated that in patients suffering from long COVID, a close eye should be kept clinically in order to diagnose at an early stage. 

### 4.2. Limitations of the Study

One of the limitations of this study was the small number of cohorts used. Out of 1980 patients based on inclusion and exclusion criteria, only 143 patients remained who could be included in the study. Furthermore, only 48 had long COVID-19. Studies on larger cohorts could help draw more accurate conclusions. Another limitation of the study was the lack of continuous glucose monitoring with the help of sensors. 

### 4.3. Ongoing Research and Future Directions

As the scientific community continues to investigate the complexities of long COVID-19, ongoing research aims to unravel the precise mechanisms underlying the connection between the lingering effects of the virus and insulin resistance. Understanding these mechanisms is crucial for developing targeted therapies and interventions to address the metabolic implications of long COVID-19.

## 5. Conclusions

Despite rigorous public health interventions and substantial vaccination coverage in certain nations, a post-COVID-19 syndrome has surfaced, characterized by an absence of a clear definition, known prevalence, or etiology. Metabolic dysfunction, including obesity, insulin resistance, and diabetes mellitus, is a known risk factor for severe acute COVID-19. Emerging evidence suggests that these factors, coupled with a chronic inflammatory state, might also increase susceptibility to complications. 

The link between long COVID-19 and insulin resistance sheds light on the diverse and often systemic consequences of SARS-CoV-2 infection, as seen in our study. Healthcare providers and researchers must collaborate to deepen our understanding of this connection and provide better care for individuals grappling with the long-term effects of the pandemic. This study concluded that the incidence increases in patients with long COVID-19. As we strive to overcome the challenges posed by long COVID-19, a comprehensive approach that considers both respiratory and metabolic aspects will be essential for improving the quality of life for those affected by this lingering condition.

## Figures and Tables

**Table 1:** General characteristics of the study lot.

	Age	Sex	HOMA iR	TyG	Glicemie To	ESR T0	CRP T0	PP	SBP	MAP	DBP	Non-HDL	Chol.	TG	HDL	LDL	Uric Acid	W	BMI
count	143.00	143.00	143.00	143.00	143.00	143.00	143.00	143.00	143.00	143.00	143.00	143.00	143.00	143.00	142.00	143.00	143.00	142.00	140.00
unique		2.00																127.00	101.00
top		m																81.40	30.70
freq		73.00																3.00	3.00
mean	55.05		4.08	2.02	105.05	45.37	32.69	55.24	142.03	105.13	86.71	168.56	217.58	195.90	49.37	129.52	5.83		
std	10.52		3.90	1.22	34.01	24.58	21.05	13.74	21.33	14.26	11.74	49.21	49.41	106.13	13.38	45.23	2.08		
min	31.00		0.57	0.45	63.10	12.00	5.00	30.00	105.00	80.00	65.00	86.90	120.00	57.20	14.00	0.00	0.00		
25%	49.50		2.18	1.13	85.30	26.00	16.00	45.00	125.00	93.33	80.00	137.65	183.55	126.50	39.90	102.09	4.90		
50%	56.00		3.07	1.69	96.10	40.00	25.00	55.00	140.00	105.00	85.00	160.50	209.00	175.30	47.00	126.70	5.70		
75%	62.00		4.71	2.37	114.45	60.00	50.00	60.00	157.50	115.83	95.00	192.10	245.65	223.65	56.95	160.07	7.10		
max	79.00		36.41	6.46	269.70	120.00	80.00	100.00	220.00	160.00	130.00	387.90	443.40	623.90	97.10	301.56	11.70		

HOMA iR: Homeostatic Model Assessment for Insulin Resistance; TyG: triglyceride—glucose index; T0: at time of admission/first presentation; ESR: erythrocyte sedimentation rate; CRP: C-reactive protein; SBP: systolic blood pressure; MAP: mean arterial pressure; DBP: diastolic pressure; HDL: high-density lipoprotein; Chol.: cholesterol; LDL: low-density lipoprotein; W: weight; BMI: body mass index.

**Table 2:** Occurrence of diabetes mellitus in obese patients after COVID-19.

	Number of Patients	Glycemia-t0 > 106 mg/dL		Glycemia-t4 > 106 mg/dL		Glycemia-t12 > 106 mg/dL	
	INIT	n	%	n	%	n	%
BMI > 30 kg/m^2^	115	42	36.52	62	53.91	72	62.61
BMI ≤ 30 kg/m^2^	28	8	28.57	10	35.71	13	46.43
TOTAL	143	50	34.97	72	50.35	85	59.44
*p*-value		0.214		0.042		0.058	

BMI: body mass index; INIT: on first admission, t0, t4, and t12: at time of admission, at 4 months, and at 12 months follow-up, respectively.

**Table 3:** Incidence of occurrence of diabetes in long COVID vs. non-long COVID patients.

Data	Number of Patients	Gycemia at t4 and t12 ≥ 106 mg/dL	%
Long-COVID-19	48	36	75.00
Non-Long-COVID-19	95	53	55.79
Total	143	88	62.24
**Statistics**			
**Difference %**	**95% CI**	**Chi sq**	**Significance *p*-Value**
19.21	2.43% to 33.42%	4.972	0.0258

t4 and t12: at 4 and 12 month follow-up, respectively; CI: confidence interval; Chi sq: chi-square.

**Table 4:** Relationship between obesity and long COVID.

	BMI—Mean Value	Standard Deviation
**Long COVID-19**	34.78	5.81
**Non-Long COVID-19**	33.74	4.27
*p*-value	0.277	>0.05

BMI: body mass index.

**Table 5:** Correlation between the inflammatory status and metabolic status after COVID-19 acute infection.

	TyG	Glycemia	Insulinemia	HOMA-IR	ESR	hs-CRP
TyG	1					
Glycemia	−0.4115	1				
Insulinemia	−0.29008	−0.06497	1			
HOMA-IR	−0.40269	0.282608	0.862863	1		
ESR	−0.38165	0.257401	0.742977	0.842319	1	
hs-CRP	−0.40288	0.224669	0.716144	0.79284	0.86634	1

TyG: triglyceride–glucose index; HOMA-IR: Homeostatic Model Assessment for Insulin Resistance; ESR: erythrocyte sedimentation rate; hs-CRP: high-sensitivity C-reactive protein.

## Data Availability

Source data are provided with this paper. All other data supporting the findings of this study are available from the corresponding author upon written request.
